# The Value of Fortified Aminoglycoside/Cephalosporin Treatment as First-Line Treatment and in Fluoroquinolone-Resistant Bacterial Keratitis

**DOI:** 10.4274/tjo.galenos.2020.37346

**Published:** 2020-10-30

**Authors:** Özlem Dikmetaş, Yağmur Deniz, Sibel Kocabeyoğlu, Merve Başol, Murat İrkeç

**Affiliations:** 1 Hacettepe University Faculty of Medicine, Department of Ophthalmology, Ankara, Turkey; 2 Hacettepe University Faculty of Medicine, Department of Biostatistics, Ankara, Turkey

**Keywords:** Bacterial keratitis, topical treatment, fortified antibiotics

## Abstract

**Objectives:**

Topical application of fluoroquinolone antibiotics is thought to be as effective as fortified antibiotics. The aim of this study was to evaluate the efficacy of fluoroquinolones as an alternative to fortified antibiotic therapies.

**Materials and Methods:**

The medical records of 31 patients who were hospitalized in our department due to bacterial keratitis were retrospectively reviewed. Fluoroquinolone was started as the first treatment for 20 (64.5%) patients and upon no response fortified antibiotic was initiated, and 11 (35.5%) patients were started with fortified treatment. Cultures and smears were recorded before treatment. Lesions were evaluated as superficial or deep according to their depth. Treatment response was evaluated based on reduction of infiltrate depth and size, change in visual acuity, and regression of hypopyon.

**Results:**

Central, paracentral, and peripheral location were detected in 9 (29.0%), 10 (32.2%) and 12 (38.7%) eyes, respectively. According to lesion depth, 15 (48.3%) were deep and 16 (51.6%) were superficial. Response of superficial lesions was found to be statistically earlier (p=0.037). Culture was positive in 9 (29.0%) eyes. The initial best corrected visual acuity (BCVA) was 0.5±0.7 logMAR (-0.1-2.3) and 0.3±0.3 logMAR (-0.1-0.9) after treatment. Treatment response showed moderate but statistically nonsignificant correlation with time to treatment initiation and initial BCVA (r=0.527, p=0.184; r=0.517, p=0.120).

**Conclusion:**

Although fluoroquinolones are the first choice for the treatment of bacterial keratitis, fortified antibiotics have been shown to be effective in patients who do not respond to treatment. Fortified therapy should be kept in mind in the treatment of bacterial keratitis.

## Introduction

Infectious keratitis is a condition characterized by uncontrolled inflammation associated with the proliferation of bacteria, viruses, fungi, or parasites in the cornea due to impaired defense mechanisms for various reasons.^1,2,3^ If not diagnosed accurately and treated early, it can result in severe vision loss.^3^ The annual incidence is 6.3-710 per 100,000, with higher rates among contact lens users.^1,4,5,6,7,8^ Although culture and smear are frequently used in the diagnosis of keratitis, accurate and rapid diagnosis is currently made with polymerase chain reaction and *in vivo* confocal microscopy.^7^

Bacterial keratitis is infectious keratitis caused by bacteria. *Staphylococcus aureus* and *Streptococcus pneumoniae*, which are frequently associated with eyelid and tear film problems, and *Pseudomonas aeruginosa*, which is frequently seen as a result of contact lens use, are common causative agents.^1^ Hospitalization of the patient may be preferable because the condition requires fast and effective treatment after diagnosis. In particular, it is more appropriate to hospitalize patients who have central corneal involvement, rapid progression, clinical signs of virulent bacteria, and those who are unlikely to have adequate care at home.^9^ The aim of treatment is to eliminate the causative agent and ensure minimal structural damage.^9^ Treatment should be started immediately after obtaining a corneal sample. Due to the possibility of rapid progression and poor prognosis, empirical antibiotic therapy should be initiated in patients those pathogen is undetermined.

Numerous antibiotics can be used in the treatment of keratitis. These antibiotics should be bactericidal and have low toxicity to ocular tissues. Therefore, fortified antibiotic combination therapies are used after analyzing their effectiveness against gram-positive and gram-negative bacteria.^9^ First-generation cephalosporins (especially cefazolin), glycopeptides (vancomycin), aminoglycosides, and fluoroquinolones are used for gram-positive bacteria, while aminoglycosides and fluoroquinolones are used for gram-negative bacteria.^10,11^ Fluoroquinolones have also been used because they act against both gram-positive and gram-negative bacteria and provide high treatment success with a single drug.^10,11^ However, although the probability of developing resistance was expected to be lower than other bacteria, resistance develops more rapidly. The most important disadvantage of fluoroquinolones was their low activity against gram-positive bacteria, especially streptococci, but this activity was improved with the development of fourth-generation fluoroquinolones.^10,11^

The current study aimed to demonstrate the effectiveness of fortified antibiotic combination therapy, which is now less preferred than fluoroquinolones.^12^ The objective was to evaluate the efficacy of this treatment in cases of bacterial keratitis initially treated with fourth-generation fluoroquinolone therapy or with fortified aminoglycoside/cephalosporin therapy.

## Materials and Methods

This study was performed after obtaining approval from the Hacettepe University Faculty of Medicine Ethics Committee (no. GO 17/264) and adhered to the principles of the Declaration of Helsinki. The medical data of patients who presented to the Cornea Unit of the Hacettepe University Faculty of Medicine Department of Ophthalmology were evaluated retrospectively. Of these patients, those who had previously started treatment, those treated at another center, those with systemic comorbidity, contact lens users, and those with other ocular surface diseases were excluded. Causes of keratitis include bullous keratopathy, recurrent corneal epithelial defect, trauma-induced epithelial defect, and blepharitis. A total of 31 patients who presented directly to our hospital, had not been treated previously, and were treated in our clinic were included in the study.

Treatment with a fourth-generation fluoroquinolone (5 mg/mL moxifloxacin) or fortified cephalosporin (50 mg/mL cefazolin) and aminoglycoside (14 mg/mL gentamicin) combination therapy was initiated. Fortified antibiotics were prepared daily for use. All patients received 1 drop every 15 minutes for the first 6 hours, hourly day and night for 48 hours, hourly during the day for the next 3 days, and tapered thereafter depending on the clinical course. Patients who did not respond to fluoroquinolone within the first 72 hours were switched to fortified antibiotic therapy.^13^ This applied to all cases. The patients were given no other treatment before these medical treatments. None of the patients received steroid therapy.

The patients’ best corrected visual acuity (BCVA), intraocular pressure, and anterior and posterior segment examination findings were evaluated. In addition, culture and smear results were analyzed. Deep and superficial corneal infiltration were differentiated based on the involvement of half or more of the full corneal thickness in the biomicroscopic examination.^14^ Treatment response was evaluated based on the reduction in the depth and size of the corneal infiltrate, regression of corneal edema, change in visual acuity, anterior chamber inflammation, and regression of hypopyon.^15^ Patients with infiltrates located in the central cornea and larger than 2 mm and all patients started on fortified antibiotic treatment were hospitalized for treatment.

### Statistical Analysis

For descriptive statistics, continuous variables were expressed as mean and standard deviation, and categorical variables as number and percentage. Categorical variables (lesion depth, hypopyon, lesion localization) were compared using chi-square test. Relationships between categorical variables and numerical variables were analyzed using eta correlation coefficient. The level of significance was accepted as p<0.05. Analyses were performed using IBM SPSS version 21.0.

## Results

At diagnosis, the mean age of the patients (18 males and 13 females) was 49.1±24.2 (3-88) years. Mean BCVA was 0.5±0.7 (-0.1-2.3) logMAR (logarithm of the minimum angle of resolution) before treatment and 0.3±0.3 logMAR (-0.1-0.9) after treatment. Subgroup analysis based on 4 age groups (0-16, 17-50, 51-80, and >81 years) revealed no significant correlation between age and initial or final BCVA (r=0.325, p=0.074; r=-0.254, p=0.201). There were no significant differences in initial or final BCVA among the age groups (p=0.695, p=0.096). Mean treatment duration was 3.2±0.3 (1-10) weeks. Three patients had short follow-up periods and it was noted that these patients had peripheral and superficial infiltrates. In these patients’ final follow-up examination, their BCVA was perfect and the lesions had resolved. The general demographic and clinical characteristics of the patients are summarized in Table 1.

For 64.5% (20/31) of the patients, fourth-generation fluoroquinolone therapy was used as first-line treatment and fortified aminoglycoside/cephalosporin treatment was initiated after no response was obtained. Of these 20 eyes, infiltrates were peripheral in 12 (60%) and paracentral in 8 (40%); none had central lesions. BCVA in these eyes was 0.3±0.2 (-0.1-0.7) logMAR before treatment and 0.2±0.3 (-0.1-0.9) logMAR after treatment. The mean follow-up period for these patients was 3.5±0.3 (3-6) weeks. In the entire study group, treatment response showed moderate but statistically nonsignificant correlation with time to treatment initiation and initial BCVA (r=0.527, p=0.184; r=0.517, p=0.120). Earlier initiation of treatment was associated with better treatment response. Patients with low initial BCVA had lower final BCVA and poor treatment response in terms of corneal infiltrates.

When corneal smear and culture results were examined, microorganisms were detected in the smears of 6 eyes (19.3%) and culture was positive in 9 eyes (29%). Of the microorganisms demonstrated, 6 (66.6%) were gram-positive bacteria and 3 (33.3%) were gram-negative bacteria; no fungi or parasites were detected (Table 2). The most common pathogen was *Staphylococcus epidermidis*, followed by *Streptococcus mitis, P. aeruginosa, Klebsiella pneumoniae*, and *Haemophilus influenzae*. According to culture results, *S. aureus* keratitis was seen in only 1 patient, whose final BCVA was lower than the initial level.

Keratitis foci were located centrally, paracentrally, and peripherally in 9 (29%), 10 (32.2%), and 12 (38.7%) of the eyes, respectively, and hypopyon was detected in 5 eyes (16.1%). Presence of hypopyon in the anterior chamber was found to be associated with poor treatment response (p=0.001). According to lesion depth, 15 (48.3%) of the lesions were deep and 16 (51.6%) were superficial. Superficial lesions showed significantly faster response to treatment (p=0.037). Three patients (9.6%) who did not respond to treatment underwent amniotic membrane transplantation. These 3 patients had BCVA of 2.3, 2.3, and 0.9 logMAR before treatment and 0.9, 0.9, and 0.9 logMAR after treatment, respectively.

## Discussion

This study demonstrated the effectiveness of fortified aminoglycoside/cephalosporin combination therapy in eyes with bacterial keratitis when used as first-line treatment or after non-response to fourth-generation fluoroquinolone therapy. Of the patients included in the study, 64.5% (20/31) were first treated with fourth-generation fluoroquinolone, while 35.5% (11/31) received fortified aminoglycoside/cephalosporin combination therapy as first-line treatment. Patients in the fluoroquinolone group who did not respond to treatment were treated with fortified aminoglycoside/cephalosporin. In total, 90.3% (28/31) of the patients responded to treatment, while 9.6% (3/31) did not. These 3 non-responders underwent amniotic membrane transplantation and their visual acuity remained stable. As for the reasons for nonresponse to treatment, deep lesions and presence of hypopyon were found to be significant in our study.

Similar to our study, Karalezli et al.^16^ administered fluoroquinolone or aminoglycoside/cephalosporin combination therapy separately to both groups and compared their efficacy, and they did not detect any statistically significant differences between these two antibiotic groups. Unlike other studies, in the present study we evaluated the outcomes of patients who were first treated with fourth-generation fluoroquinolone and switched to fortified aminoglycoside/cephalosporin combination therapy after non-response to treatment, compared to patients who used fortified aminoglycoside/cephalosporin combination as first-line treatment.

Because bacterial keratitis can result in severe vision loss, empirical antibiotic treatment should be initiated early, without waiting for culture and smear results.^15^ Although the culture positivity rate in keratitis varies in studies conducted worldwide, the mean rate is around 30-50%.^17,18^ In our study, the positive culture rate was 29% (9/31).

Broad-spectrum antibiotic monotherapy has gained popularity due both to its practicality and the notion that administering a single drug will reduce adverse effects. Fourth-generation fluoroquinolones are frequently used for this purpose. Fluoroquinolone formulations are also preferred as monotherapy due to their broad-spectrum activity, stability at room temperature, convenience for patients, low cost, and solution stability features.^19^ Fluoroquinolones are therapeutic agents with very good tissue penetration and the least ocular toxicity.^20^ The main problem with drugs applied to the ocular surface is being able to reach the effective dose in the cornea. Topical agents may have low bioavailability for this reason. The mucoadhesive polymeric hydrogel formulations used with fluoroquinolones facilitate the drug reaching the therapeutic dose in the cornea.^21^ They exert their effect by inhibiting bacterial DNA synthesis.^10^

While first-generation fluoroquinolones mainly act against gram-negative bacteria, new-generation fluoroquinolones have increased activity against gram-positive bacteria, but their effectiveness against *Pseudomonas* strains could not be increased. At present, the most effective fluoroquinolone against *Pseudomonas* strains is ciprofloxacin, a second-generation fluoroquinolone.^22^ Kowalski et al.^23^ showed that moxifloxacin and gatifloxacin, both fourth-generation fluoroquinolones, were more effective against gram-positive and gram-negative bacteria, respectively, compared to other generations of fluoroquinolones.

Previous studies show that despite their effectiveness, the development of resistance against fluoroquinolones has become an important problem.^24,25^ With this group of antibiotics, sufficient gram-positive/gram-negative activity cannot be achieved against all microorganisms when administered alone and resistance may develop quickly.^26^ Due to differing effects of fluoroquinolones against gram-negative and gram-positive bacteria and the important problem of antibiotic resistance, the known effectiveness of fortified aminoglycoside/cephalosporin combination antibiotics is still preferable, as our study also suggests.

Aminoglycosides are mainly effective against gram-negative bacteria and inhibit protein synthesis by binding to the 30S subunit of bacterial ribosomes.^27^ Although gentamicin is frequently used, tobramycin and amikacin may be preferred in case of resistance. Tobramycin in particular is an important option from the aminoglycoside group of drugs that is preferred for its marked effectiveness against *P. aeruginosa*.^27^ Aminoglycosides are often combined with beta-lactam antibiotics to increase their bactericidal activity.^27^ Cephalosporins are a group of antibiotics related to beta-lactams that show a dose-dependent effect by inhibiting cell wall synthesis.^28^ They act against both gram-positive and gram-negative bacteria. Fourth-generation cephalosporins in particular have a broad spectrum of activity and may be preferable in patients with antibiotic resistance.

Hanet et al.^11^ conducted a literature review analyzing 8 randomized and 5 nonrandomized studies and in their comparison of fluoroquinolones and fortified antibiotics, they demonstrated fluoroquinolones is appropriate as an alternative, second-line treatment option to fortified antibiotics. Constantinou et al.^29^ found that fortified antibiotic treatment and second-generation fluoroquinolone-derivative antibiotics were similarly effective. Unlike our study, these studies directly compare two different treatment methods. However, in our study we evaluated the effectiveness of fortified aminoglycoside/cephalosporin after nonresponse to fluoroquinolones in one group. Based on this, fortified antibiotics may be a preferable option, especially to prevent the problem of antibiotic resistance.

In our study, we also observed that most keratitis patients who did not respond to initial treatment with fluoroquinolone responded to fortified antibiotics. Patients whose treatment was started early had better final visual acuity and corneal infiltrate response to treatment. This may be related to the fact that in patients who presented earlier, corneal lesions induced less inflammatory response during this period.

Sharma et al.^30^ also compared the efficacy of gatifloxacin and tobramycin-cefazolin fortified antibiotic therapy in keratitis eyes and reported that they were equally effective and not substantially different in cost in developed countries. However, despite the equal effectiveness in these studies, the preference of new generation fluoroquinolone-derivative agents as first-line treatment should be limited due to resistance. Our study showed that fortified therapy was effective in cases of bacterial keratitis that were unresponsive to fourth-generation fluoroquinolones and those initially treated with fortified aminoglycoside/cephalosporin combination. In patients who do not respond to fluoroquinolones, fortified antibiotic therapy should be considered as an option.

### Study Limitations

The main limitations of this study are its retrospective design, absence of a control group, low culture positivity rate, inability to evaluate treatment adherence in patients not hospitalized during fluoroquinolone treatment, and not performing drug stability assessment.

## Conclusion

In light of the studies in the literature, we conclude that fortified antibiotics still have a place in the treatment of bacterial keratitis and remain the best alternative to fluoroquinolone therapy. This study emphasizes that fortified antibiotic therapy must be kept in mind and its effectiveness not forgotten.

## Figures and Tables

**Table 1:**
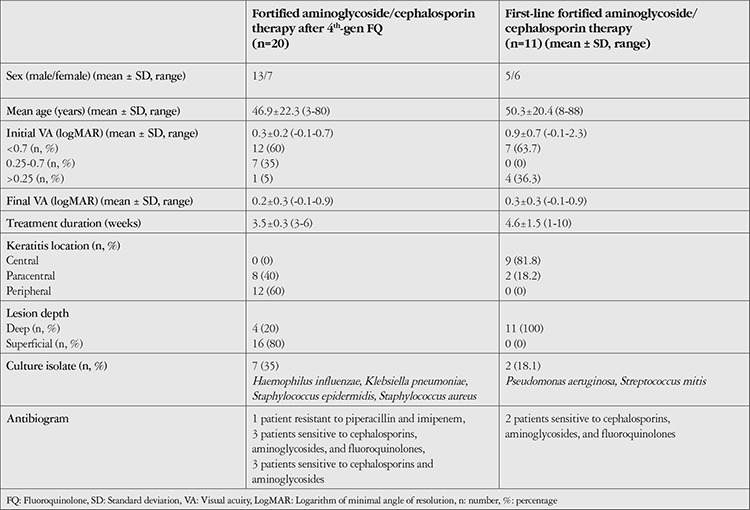
Demographic and clinical characteristics

**Table 2:**
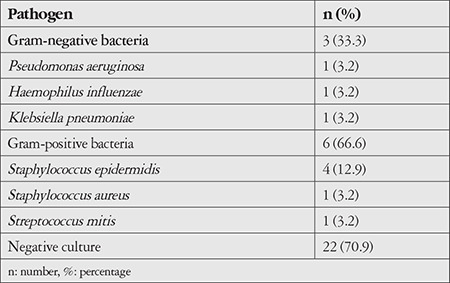
Culture results
